# Screening for TB in Hospitalised Patients with Inflammatory Bowel Disease before Anti-TNF Therapy: Is QuantiFERON^®^ Gold Testing Useful?

**DOI:** 10.3390/jcm10091816

**Published:** 2021-04-21

**Authors:** Jessica Lovatt, Deborah Gascoyne-Binzi, Thomas Hussey, Maya Garside, Fiona McGill, Christian P. Selinger

**Affiliations:** 1 Gastroenterology, Leeds Teaching Hospitals NHS Trust, Leeds LS1 3EX, UK; jessicalovatt@nhs.net (J.L.); maya.garside2@nhs.net (M.G.); 2 Laboratory Sciences, Leeds Teaching Hospitals NHS Trust, Leeds LS1 3EX, UK; deborah.gascoyne-binzi@nhs.net; 3 Anaesthetics, Bradford Teaching Hospitals NHS Foundation Trust, Bradford BD9 6RJ, UK; tomhussey@doctors.org.uk; 4 Infectious Diseases, Leeds Teaching Hospitals NHS Trust, Leeds LS1 3EX, UK; f.mcgill@nhs.net; 5 Medical Microbiology, Leeds Teaching Hospitals NHS Trust, Leeds LS1 3EX, UK; 6 Leeds Institute of Medical Research at St James’s, University of Leeds, Leeds LS2 9JT, UK

**Keywords:** inflammatory bowel disease, latent tuberculosis, QuantiFERON**^®^**

## Abstract

Background—Tumour necrosis factor alpha (TNFα) plays an important role in the pathogenesis of inflammatory bowel disease (IBD) and in immunity to *Mycobacterium tuberculosis.* Patients should be tested for latent tuberculosis infection using interferon-gamma release assays (IGRA/QF) prior to anti-TNFα therapy. Indeterminate QF results can delay anti-TNFα therapy. We sought to investigate factors associated with indeterminate QF results. Method—Retrospective study of all IGRA tests requested for gastroenterology patients in 2017. We compared inpatients and outpatients and investigated factors potentially associated with QF usefulness (steroid exposure, C-reactive protein (CRP), hypoalbuminaemia, thrombophilia). Results—We included 286 outpatients and 74 inpatients with IBD. Significantly more inpatients had an indeterminate IGRA (52.7% vs. 3.14% in outpatients; *p* < 0.0001). Laboratory parameters reflecting inflammation (high CRP, low albumin, low haemoglobin and high platelets) were also associated with an indeterminate QF (*p* < 0.0001). Exposure to steroids was more common in patients with an indeterminate QF (*p* < 0.0001). A binary logistic regression analysis revealed inpatient status and steroid exposure to be independently predictive of an indeterminate QF (*p* < 0.0001). Conclusion—There is a high chance of indeterminate QF results in inpatients. QF testing should ideally be performed in the outpatient setting at diagnosis.

## 1. Introduction

Inflammatory bowel disease (IBD), which incorporates both Crohn’s disease and ulcerative colitis, is a chronic, relapsing and remitting inflammatory condition affecting the gastrointestinal tract [1]. The pathogenesis of IBD is not fully understood and likely to be multifactorial [2]. A well accepted theory is that of the abnormal immune response to gastrointestinal flora, which leads to an immune mediated inflammation of the intestinal tract. The role of tumour necrosis factor alpha (TNFα), a pro-inflammatory cytokine, is well recognised in the pathogenesis of IBD [2].

TNFα is produced by monocytes, macrophages and T lymphocytes, and several other cell types, and can be released in response to multiple stimuli, including bacteria, viruses, immune complexes and stress [3]. It is a highly pro-inflammatory cytokine which is involved in the activation of granulocytes and T cells and the induction of fever, amongst many other roles [4]. Once released, TNFα binds to two distinct receptors to exert its pro-inflammatory effect by increasing the production of IL-1β and IL-6, causing proliferation of pro-coagulant factors and fibroblasts and initiating acute phase responses [5].

The aetiology of IBD is thought to be due to an abnormal immune response alongside environmental triggers, where the luminal epithelial barrier is breached, leading to a cascade of pro-inflammatory responses [6]. It has been established that IBD is associated with a T-cell mediated response facilitated by TNFα, as well as interleukin-12 and interferon gamma [6]. These findings, therefore, lead to the identification of TNFα as a potential therapeutic target in the management of IBD.

Anti-TNFα therapies have multiple mechanisms of action, working by reversing the aforementioned pro-inflammatory effects. They have been shown to reduce overexpression of TNFα, induce reverse signalling, where anti TNFα actually binds to a precursor of TNFα, leading to cytokine suppression [7] and induce apoptosis of T-lymphocytes [8]. Anti-TNFα therapies are highly effective agents for the treatment of IBD, and include Infliximab (UC, CD), Adalimumab (UC, CD), Golimumab (UC) and Certolizumab (CD in US and selected European countries only) [9].

TNFα also plays a vital role in host immunity following the initial infection with *Mycobacterium tuberculosis*. It is thought that the suppression of TNFα inhibits the walling off of *Mycobacterium tuberculosis* [10], thus posing a risk of re-activation of latent tuberculosis infection (LTBI). As a result, practice in most centres is to screen patients for LTBI prior to commencing anti-TNFα therapy. Tuberculin skin tests are not preferred because of false positive results due to BCG vaccination, and they require a second visit to read and interpret the result. Interferon-gamma releasing assays (IGRAs), such as QuantiFERON^®^ TB Gold In-Tube (Qiagen, Hilden, Germany) and T-SPOT.TB (Oxford Immunotec, Oxford, UK), are more specific and only require one clinic visit.

IGRAs work by measuring the T-cell immune response to TB specific antigens, and require an adequate host immune response to yield a valid result [11]. In our centre, at the time of this study, routine screening for LTBI prior to treatment with anti-TNFα agents was performed with QuantiFERON^®^ TB Gold In-Tube. Blood is incubated in tubes coated with TB peptide antigens and interferon gamma is released from activated lymphocytes in patients who have previously been infected with *Mycobacterium tuberculosis*. An indeterminate response can occur if there is a suboptimal immune response, excess background interferon gamma, or where the sample has not been adequately mixed on collection or incubated appropriately. This means the test returns a result that cannot be interpreted safely and is hence classified as indeterminate. If an indeterminate result is recorded which is due to a suboptimal immune response, our practice is to perform a subsequent T-SPOT.TB test. The T-SPOT.TB test works by isolating the lymphocytes from a blood sample so that a standardised number of cells can be added to the test. The number of cells that produce gamma-interferon are then counted. Theoretically, this could be more sensitive as, if required, the cells can be isolated from a larger volume of blood to obtain the correct concentration. QuantiFERON^®^ TB looks for the increase in the concentration of gamma interferon in 1 mL of blood.

We noticed a high rate of indeterminate QuantiFERON^®^ TB Gold In-Tube results in our inpatients who were tested during an acute flare of IBD, prior to commencing anti-TNFα, and sought to confirm whether an acute flare of IBD requiring inpatient treatment was associated with an indeterminate result. We compared blood test results to identify whether these could help predict the outcomes of QuantiFERON^®^ TB Gold In-Tube tests, and whether the dose of, or time on, steroids had an impact. We compared the inpatient population against the outpatient IBD patients, who tend to be less acutely unwell and, therefore, tests are usually requested outside a period of an acute IBD flare.

## 2. Materials and Methods

This was a single centre, observational, retrospective study. The data were collected from Leeds Teaching Hospital Trust (LTHT), a secondary and tertiary care centre in West Yorkshire with a large gastroenterology department and specialist IBD team providing care for 4000 patients with IBD.

All QuantiFERON^®^ TB Gold In-Tube samples requested by a Gastroenterology consultant from either the Gastroenterology inpatient wards or the assessment unit, or from outpatients’ department between 1 January 2017 and 31 December 2017, were included. Hospital electronic records were then used to collect patient level data on in- or outpatient status, steroid exposure and laboratory parameters. Our routine IGRA testing changed in 2019 to QuantiFERON^®^ TB Gold Plus. In addition, we therefore assessed a further cohort of patients from 2019, meeting the same criteria of those who underwent QuantiFERON^®^ TB Gold Plus tests.

QuantiFERON^®^ TB Gold In-Tube and QuantiFERON^®^ TB Gold Plus were requested when patients were considered to potentially require biological therapy. Samples were processed according to the manufacturer’s instructions. An indeterminate result was reported when, due to a poor immune response, the mitogen (positive control) value was less than 0.5 IU/mL, or when, due to a high background level of interferon gamma, the test value was >8 IU/mL.

The TB incidence in Leeds was 8.3 per 100,000 in 2017. However, neighbouring Bradford and Kirklees had much higher incidence rates of 16.6 and 10.5 per 100,000 respectively.

### 2.1. Statistical Analysis

Statistical analysis was performed using IBM SPSS Statistics 25. Categorical data were compared using Pearson’s chi-squared and Fisher’s exact tests. Continuous data were compared using independent samples of t-test or Mann–Whitney U-test, as appropriate. Factors meeting criteria for significance (*p* < 0.05) were considered for multivariate analysis. Where factors were thought to represent the same physiological entity (for example, markers of inflammation such as CRP, albumin, platelets), only one was entered to ensure a valid model for the binary logistic regression analysis (mode enter).

### 2.2. Ethics Approval and Consent Exemption

The study was conducted as a retrospective clinical service evaluation. Relevant authorisation was obtained. Due to the nature of this pragmatic service evaluation study, research ethics committee approval and informed consent were not required [12].

## 3. Results

### 3.1. Inpatients

We identified 91 inpatients who had a QuantiFERON^®^ TB Gold In-Tube test sent as part of their inpatient stay under gastroenterology in 2017. After excluding 17 patients not meeting study criteria, 74 inpatients—46% of whom were female (*n* = 34)—all actively managed for an acute flare of IBD (Table 1) were included. Of these, 43 had a diagnosis of UC, 22 of CD and the remaining 9 either IBD-U or inconclusive histology. A total of 39 (53%) patients who were tested for latent TB during their inpatient stay returned an indeterminate QuantiFERON^®^ TB Gold In-Tube. Of these 39 inpatients, 38 were being treated with IV hydrocortisone therapy. Nine patients had been on IV steroid therapy for <24 h (24%), seven had had between 24–48 h of IV steroids (18%), and 22 patients had been on IV steroid therapy for longer than 48 h (58%) at the time of QuantiFERON^®^ sampling.

The remaining 35 patients (47%) tested negative for latent TB via the QuantiFERON^®^ TB Gold In-Tube. A total of 34 (97%) patients were on IV steroids; 1 patient had a negative result and was not on any steroids. Of the 34, 11 (32%) had been on IV steroids for less than 24 h, 6 (18%) had been on IV steroids for 24–48 h, and 17 (50%) had been on steroids for longer than 48 h. None of the inpatients tested positive for Latent TB. A summary and comparison of the inpatients can be seen in Table 1.

To determine whether levels of inflammatory response or acuity of the illness are associated with the likelihood of an indeterminate result, we analysed markers of inflammation. Of the indeterminate results, 32 (82%) had an elevated CRP (>10); 38 (97%) had a low serum albumin (<35); anaemia was present in 26 patients (67%); and thrombocythemia was found in 27 (69%) patients. In comparison, the patients whose QuantiFERON^®^ TB Gold In-Tube results were negative tended to show a less severe inflammatory response. A total of 25 (71%) had an elevated CRP; 29 (82%) were hypoalbuminaemic; 19 patients (54%) were anaemic; and 10 patients were thrombocythemic (29%). Thrombocythaemia, however, was the only statistically significant comparison (*p* = 0.0006)

### 3.2. Outpatients

A total of 287 patients who attended an IBD outpatient appointment were tested for latent TB using QuantiFERON^®^ TB Gold In-Tube in 2017. Ten patients (3%) tests were returned as indeterminate. Eleven patients were tested as positive (4%), and 266 patients returned a negative result (93%) (Table 2). Of the outpatient cohort, one patient had to be excluded from further analysis, as there was no letter from a clinic detailing whether they were on steroids. Of the 286 included outpatients, 173 had a diagnosis of CD, 86 of UC, and 17 had IBD-U or inconclusive histology.

The blood results of the outpatients were also recorded. It should be noted here that not all outpatients had a full set of bloods taken in addition to the QuantiFERON^®^ TB Gold In-Tube test. For clarity, in Table 3, the total number of patients who had certain blood tests is stated at the top of each different blood test row.

### 3.3. Factors Associated with an Indeterminate QuantiFERON^®^

To determine which factors were associated with an indeterminate QuantiFERON^®^ result, we compared those with an indeterminate result with those that gave a clear positive or negative reading. Age and sex were not associated with an indeterminate QuantiFERON^®^ result (Table 3). Inpatients, patients exposed to steroids, those exposed to intravenous rather than oral steroids, and those on IV steroids longer than 24 h were more likely to have an indeterminate QuantiFERON^®^ result. Several laboratory parameters reflecting inflammatory states were associated with an indeterminate QuantiFERON^®^ result, including higher CRP, lower albumin, lower haemoglobin, and higher platelets. Several factors associated with an indeterminate QuantiFERON^®^ result belong to only three themes (laboratory parameters of inflammation, inpatient status and steroid exposure). To ensure a valid model we only included one factor from each theme in binary logistic regression analysis (Table 4). Inpatient status and steroid exposure were both independently associated with an indeterminate QuantiFERON^®^ result.

### 3.4. Comparison of Inpatient vs. Outpatients

Significantly more inpatients had an indeterminate QuantiFERON^®^ TB Gold In-Tube result (52.7% vs. 3.14% in outpatients; *p* < 0.0001). There were significant differences between in- and outpatients regarding factors potentially affecting IGRA usefulness. A total 73 (98.6%) inpatients were on high dose steroids when they were screened for latent TB compared to 68 (23.77%) outpatients (*p* < 0.0001). Of inpatients, 57 (77.02%) had a raised C-reactive protein (CRP) compared to 74 (31.49%) outpatients (*p* < 0.0001). A total of 68 (91.89%) inpatients had low serum albumin compared to 59 (25.10%) outpatients (*p* < 0.0001). Thrombocytophilia occurred in 37 (50%) inpatients and 43 (18.07%) outpatients (*p* < 0.0001).

### 3.5. Comparison of QuantiFERON^®^ TB Gold In-Tube vs. QuantiFERON^®^ TB Gold Plus

At the start of 2019, we changed our first line IGRA testing from QuantiFERON^®^ TB Gold In-Tube to QuantiFERON^®^ TB Gold Plus. We performed an analysis of all IGRA tests performed for the gastroenterology department in 2019 to assess the performance of QuantiFERON^®^ TB Gold Plus (2019 cohort) in inpatients vs. outpatients, as well as to compare performance of inpatients to QuantiFERON^®^ TB Gold In-Tube (2017 cohort). In 2019, indeterminate QuantiFERON^®^ TB Gold Plus occurred in 37 of 107 inpatients (34.6%) and in 16 of 236 outpatients (6.8%; *p* < 0.0001). The proportion of inpatients testing indeterminate reduced significantly from 52.7% in 2017 (QuantiFERON^®^ TB Gold In-Tube) to 34.6% in 2019 (QuantiFERON^®^ TB Gold Plus; *p* = 0.02).

### 3.6. T-SPOT.TB Testing

Of the 39 inpatients whose QuantiFERON**^®^** results were indeterminate, 23 (59%) never had a T-SPOT.TB sent and 16 (41%) had a subsequent T-SPOT.TB taken, 11 (28%) of which were negative and 5 (13%) of which were taken but had no result; this was due to insufficient sample volume or the sample not reaching the lab in time to be sent to the testing laboratory. Of the 10 outpatients who had an indeterminate QuantiFERON^®^ result, seven had negative T-SPOT.TB results and three never had a T-SPOT.TB test undertaken.

### 3.7. Anti-TNFα Therapy

To understand subsequent treatment decisions, we reviewed the inpatient records. Five inpatients received anti-TNFα therapy following a negative T-SPOT.TB result and two patients had samples collected for a T-SPOT.TB assay; however, due to sample problems, some received anti-TNFα therapy without further delay following a clinical risk vs. benefit decision. Six inpatients received anti-TNFα therapy based on a clinical risk vs. benefit decision, and no blood was collected for T-SPOT.TB assays.

### 3.8. Development of Symptomatic TB Infections

Of 10 outpatients diagnosed with latent TB based on a positive QuantiFERON**^®^** result, 6 completed anti-tuberculous therapy, 2 declined anti-tuberculous therapy, and, in 2 cases, the respiratory physicians felt no indication for treatment. Where anti-tuberculous therapy was commenced, the treatment intent was to allow for potential anti-TNF therapy. In the end, none of these patients required treatment with an anti-TNF agent for IBD. One inpatient with latent TB completed anti-tuberculous therapy. None of these patients went on to develop symptomatic TB.

Two patients with negative QuantiFERON**^®^** results were treated with anti-tuberculous therapy. The first one was felt to be at high risk of latent TB despite a negative QuantiFERON**^®^** result, and the second one was treated for suspected abdominal TB but, unfortunately, moved out of area before treatment was completed.

## 4. Discussion

Anti- TNFα therapy has become a vital tool in the management of patients with IBD, and it is well accepted internationally that, prior to commencing therapy, screening for LTBI should be performed. This should include a thorough history to elicit any significant risk factors (ethnicity, travel history, occupation and comorbidities), alongside laboratory-based testing. In 2017, our centre used QuantiFERON^®^ TB Gold In-Tube testing; however, this can be returned as an indeterminate result which can lead to treatment delays. If an indeterminate test is returned, then the practice is to send a blood sample for a T-SPOT.TB test, which further delays treatment. Delays in Anti-TNFα-therapy are associated with poorer outcomes, highlighting the importance of reducing diagnostic delays.

Our results found that a significantly high proportion of inpatients with IBD had an indeterminate QuantiFERON^®^ TB Gold In-Tube result in comparison to the outpatient cohort (*p* < 0.0001). Furthermore, we have shown that this particular group of patients had high rates of corticosteroid use and had signs of systemic inflammation, with raised inflammatory markers corresponding to the fact that they had an ongoing acute flare. All but one of the indeterminate QuantiFERON**^®^** results were due to an inadequate immune response, suggesting that it is the immunosuppressive therapy leading to the indeterminate results rather than the acute flare itself. These findings are in keeping with other key studies [13,14]. In our centre, QuantiFERON^®^ TB Gold In-Tube takes, on average, 2–3 days to return a result; therefore, it can result in significant treatment delays. This delay is longer if the QuantiFERON^®^ TB Gold In-Tube result is indeterminate. We therefore conclude that testing for LTBI during an acute flare of IBD is an inappropriate use of resources, and it should be done earlier in the course of the disease, if possible.

A systematic review showed a sensitivity of T-SPOT.TB of 90% compared to 78% for QuantiFERON**^®^** Gold, with a specificity of 99% and 93%, respectively (however, studies with immunocompromised patients were excluded) [15]. However, in many centres, T-SPOT.TB testing is not performed on site and it can therefore take 4–5 days to return a result, which can lead to a delay in definitive anti-TNFα treatment. The cost of QuantiFERON^®^ TB Gold In-Tube is also lower in our centre compared to T-SPOT.TB.

In the general population, studies show rates of indeterminate results of 3.8% (8). However, two separate studies in populations with IBD, who were tested prior to initiation of anti-TNFα, showed indeterminate results in 19.5% and 22% of the population [13,14], where authors found corticosteroid use and hospitalization were risk factors associated with an indeterminate result.

Findings in the inpatient group are also backed up by the data collected from patients in the outpatient setting. Of these, the inflammatory markers were found to be, in general, lower or normal, indicating no active flare, and the use of corticosteroids was much lower. Consequently, the rates of indeterminate results were far lower, and the data supports the use of QuantiFERON^®^ TB Gold In-Tube in this group, particularly as there is no time pressure in the rare case that such a result is found. This provides strong evidence for the argument that all patients should be screened in the outpatient setting as soon as practicably possible.

Unfortunately, this is not possible for all patients, as some will present with an acute flare requiring hospital admission as their first presentation and, therefore, urgent workup for LTBI, without the chance for testing in the outpatient setting. As discussed, our practice is to send a sample for a T-SPOT.TB test in those patients returning an indeterminate QuantiFERON^®^ TB Gold In-Tube. In some patients, it may be more appropriate to request a T-SPOT.TB test in these circumstances. For a complete assessment for potential, it is important to include a chest X-ray and a history eliciting risk factors for latent TB. These should always be used in conjunction with QuantiFERON^®^ TB Gold In-Tube or T-SPOT.TB tests.

The literature shows conflicting results when comparing the efficacy of T-SPOT.TB to QuantiFERON^®^ TB Gold In-Tube. Overall, a meta-analysis, including 9 QuantiFERON^®^ TB Gold In-Tube studies and 12 TSPOT studies, showed no significant difference on sensitivity and specificity between the two tests [16]. However, factors such as immunosuppression and age have been shown to cause significant difference in results; although, again, studies have shown contradictory results [17,18]. What is more, many of these studies are testing patients with presumed active TB as opposed to LTBI. One centre, which uses an initial QuantiFERON^®^ TB Gold In-Tube combined with TST, used T-SPOT.TB when indeterminate results were returned, and found that T-SPOT.TB was useful in clarifying these results; however the study included only low patient numbers [19]. A further study found that indeterminate results were much lower in the TSPOT.TB (1.9%) than QuantiFERON^®^ (7.8%) [20]. These findings support the use of TSPOT.TB in patients whose QuantiFERON^®^ returns an indeterminate result. That said, overall, there is little evidence to support one over the other.

In our study, we found that T-SPOT.TB tests were actually only sent in less than half of the inpatients with indeterminate QuantiFERON^®^ TB Gold In-Tube results, but in 70% of the outpatients, again eluding the fact that outpatient management is less time pressured. All of the indeterminate results, if they were-tested for TSPOT.TB, returned negative results. Although cost effectiveness has not been looked into directly in this review, it must be taken into consideration. In our study, over half of the inpatients tested had an indeterminate result and, therefore, required a further test T-SPOT.TB. This strategy has a high cost associated with the cumulative cost of both tests, potential delays in anti TNFα therapy commencement, and associated additional hospital bed days. Given current turnaround times for T-SPOT.TB testing, the results would likely not arrive in time for the crucial third day review in patients with acute severe colitis.

Further research is clearly needed in this area to compare both the sensitivity and accuracy of the two tests—including the newer, more sensitive QuantiFERON^®^ TB Gold Plus, currently used for screening for latent TB in the IBD population—but also with reference to cost effectiveness. Reassuringly, our current test strategy has not led to any cases of activation of latent TB. However, further improvements to the current test strategy should be made.

We would suggest that, in ideal practice, patients are screened for latent TB in the outpatient setting using a QuantiFERON^®^ TB Gold In-Tube or QuantiFERON^®^ TB Gold Plus as the initial test, as this test is cheaper and yields very few indeterminate results in this population. If this is not possible, as the patient’s first presentation is an acute flare, we would suggest testing using a T-SPOT.TB assay as the primary screening test in centres where results are available in an acceptable time frame. Based on our data, and although the numbers are small, all the inpatients who had indeterminate QuantiFERON^®^ TB Gold In-Tube tests who were subsequently tested for T-SPOT.TB had negative results. By using this screening method in the first instance, delays to treatment may be reduced. Further studies following up these patients with regard to the development of TB are clearly needed, and physicians should be aware of the possibility that immunocompromised patients should be routinely questioned about symptoms of TB. There is limited research comparing the two screening tests—QuantiFERON^®^ TB Gold In-Tube and TSPOT.TB in immunocompromised patients.

As the newer version of the QuantiFERON Gold test (QuantiFERON^®^ TB Gold Plus) may have better accuracy, we analysed a cohort for 2019 where we exclusively used QuantiFERON^®^ TB Gold Plus. We saw a reduction of 34.6% in the number of indeterminate tests being returned. However, the higher rate for inpatients vs. outpatients persisted, and the rate of 34.6% remains too high to be useful in the acute ward setting.

There are a number of limitations to our study. The retrospective nature of our review meant that only data fields captured in the electronic notes or patient letters could be recorded. We did not recall all of the written paper notes for the patients in our study, so we relied on documentation online and via letters; this obviously means certain documentation could have been missed, such as any IV steroids given in the emergency department, as they do not use electronic medication charts. Furthermore, we had no access to data on other risk factors for latent TB. Our paper reports associations, and firm conclusions on causation cannot be drawn.

## 5. Conclusions

In conclusion, we recommend QuantiFERON^®^ TB Gold In-Tube/PLUS testing for LTBI at diagnosis in the outpatient setting. The acute inflammatory state of IBD seen in inpatients and steroid therapy can lead to indeterminate IGRA results that can complicate IBD management. Where clinicians encounter indeterminate QuantiFERON^®^ TB Gold In-Tube/PLUS results, and urgent anti-TNF therapy is required, a clinical risk assessment should be applied to allow therapy as clinically indicated.

## Figures and Tables

**Table 1:** Inpatient IBD patients tested for latent TB—characteristics and steroid use.

	Indeterminate QuantiFERON^®^	Negative QuantiFERON^®^
Total	39 (%)	35 (%)
Male	20 (51)	20 (57)
Female	19 (49)	15 (43)
Ulcerative Colitis	21 (54)	22 (63)
Crohn’s disease	12 (31)	10 (29)
IBD-U	4 (10)	2 (6)
Preadmission Medication		
None	24 (62)	21 (60)
Mesalazine	11 (28)	13 (33)
Immunomodulator	4 (10)	6 (17)
Biologic	0	3 (10)
On IV steroids (Hydrocortisone)	38 (97)	34 (97)
For <24 h	9 (24)	11 (32)
For 24–48 h	7 (18)	6 (18)
For >48 h	22 (58)	17 (50)
On oral steroid (Prednisolone)	1 (3)	0
<20 mg	0	-
>20 mg	1	-
On no steroids	0	1

**Table 2:** Outpatient IBD patients tested for latent TB—characteristics.

	Indeterminate QuantiFERON^®^	Negative QuantiFERON^®^	Positive QuantiFERON^®^
Total	10 (3)	265 (93)	11 (4)
Male	6 (60)	137 (52)	8 (73)
Female	4 (40)	129 (48)	3 (27)
Ulcerative Colitis	5 (50)	81 (31)	0
Crohn’s disease	4 (40)	163 (61)	6 (55)
IBD-U	1 (10)	21 (8)	5 (45)
Medication			
None	5 (50)	126 (48)	8 (73)
Mesalazine	4 (40)	88 (33)	0
Immunomodulator	1 (10)	72 (27)	2 (18))
Biologic	0	5 (2)	1 (9)
On oral steroid (Prednisolone) <20 mg >20 mg	1 (10) 6 (60)	35 (13) 18 (7)	2 (18)
On oral steroid (Budesonide)		7 (3)	
On no steroids	3 (30)	205 (77)	9 (82)

**Table 3:** Factors associated with an indeterminate QuantiFERON**^®^** Gold-in-Tube reading.

Variable	Indeterminate Result	Positive or Negative Result	Univariate Analysis *p*-Value
Sex	23 females (48%) 25 males (52%)	158 females (48%) 170 males (52%)	*p* = 0.97
Age (mean)	41.4 years	42.5 years	*p* = 0.66
Inpatient vs. outpatient	38 inpatients (79%) 10 outpatient (21%)	51 inpatients (16%) 277 outpatients (84%)	*p* < 0.0001
Steroids	45 exposed (94%) 3 not exposed (6%)	98 exposed (30%) 230 not exposed (70%)	*p* < 0.0001
Steroid route	37 intravenous hydrocortisone (82%) 8 oral prednisolone (18%)	36 intravenous hydrocortisone (39%) 56 oral prednisolone (61%)	*p* < 0.0001
Prednisolone dose (mean)	27.1 mg	19.6 mg	*p* = 0.075
Time of IV hydrocortisone exposure prior to sample drawn	0 no prior exposure 9 exposure <24 h 7 exposure 24–48 h 22 exposure >48 h	15 no prior exposure 11 exposure <24 h 9 exposure 24–48 h 16 exposure >48 h	*p* = 0.002
CRP (median)	29.5 mcg/ml	18.05 mcg/ml	*p* < 0.0001
Albumin (mean)	28.0 g/dL	35.6 g/dL	*p* < 0.0001
Haemoglobin (mean)	117.5 g/L	133.4 g/L	*p* < 0.0001
Platelets (mean)	464 × 10^9^ per liter	327 × 10^9^ per liter	*p* < 0.0001
White cell count (mean)	11.1 × 10^9^ per liter	9.0 × 10^9^ per liter	*p* = 0.29

**Table 4:** Multivariate analysis of factors associated with an indeterminate QuantiFERON^®^ Gold reading.

Variable	Categorised as	B-Value (SE)	
Inpatient vs. outpatient	Inpatient vs. outpatient	−2.637 (0.649)	*p* < 0.0001
Steroids	Exposed vs. not exposed	−1.649 (0.449)	*p* < 0.0001
CRP (median)	<20 mcg/mL vs. ≥20 mcg/mL	0.399 (400)	*p* = 0.32

## Data Availability

No raw data are available.
